# Legal and Ethical Challenges in Integrating AI Into Clinical Practice: Qualitative Study of Physicians’ Real-World Experiences

**DOI:** 10.2196/82351

**Published:** 2026-03-31

**Authors:** Mehrnaz Mostafapour, Jacqueline Fortier, Karen Pacheco, Heather Murray, Gary Garber

**Affiliations:** 1 The Canadian Medical Protective Association, 875 Carling Ave Suite 323, Ottawa, ON, K1S 5P1, Canada, 1 (613) 725-2000; 2 Department of Emergency Medicine, Queen's University, Kingston, ON, Canada; 3 Department of Medicine and the School of Public Health and Epidemiology, Faculty of Medicine, University of Ottawa, Ottawa, ON, Canada

**Keywords:** artificial intelligence, AI, medicolegal, ethics, physicians, clinical practice

## Abstract

**Background:**

The adoption of artificial intelligence (AI) in health care has accelerated; however, physicians continue to face substantial legal, ethical, and regulatory uncertainties when considering AI integration into clinical practice. Although the literature on AI in health care is expanding, there is limited insight into the real-world concerns voiced by clinicians navigating these uncharted territories.

**Objective:**

This study aimed to explore the legal and ethical uncertainties raised by Canadian physicians in relation to AI use in clinical care, using actual medicolegal advice requests as a window into their practical concerns.

**Methods:**

We conducted a comprehensive thematic analysis of 46 medicolegal advice cases made by physicians to a national medicolegal advisory service between March 2023 and February 2025. The cases were analyzed to identify key themes and patterns in physicians’ questions and perceived risks regarding AI tools in clinical contexts.

**Results:**

Eight key themes emerged, including the use of AI scribes, data privacy and security, patient consent, data ownership, regulatory uncertainty, medicolegal liability, vendor agreements, and concerns about accuracy and bias. Many of the inquiries focused on administrative and documentation-related AI applications rather than on diagnostic tools, reflecting the current stage of AI integration in everyday clinical workflows. Physicians expressed uncertainty regarding legal responsibility, alignment with privacy laws, and appropriate communication with patients about AI use.

**Conclusions:**

This study offers unique insight into frontline physicians’ real-time concerns about AI, highlighting the need for clearer regulatory guidance, clinical standards, and legal frameworks to support safe and ethical AI adoption in health care.

## Introduction

The integration of artificial intelligence (AI) into health care represents a major shift, offering the potential to improve diagnostic accuracy, enhance efficiency, and support more personalized care [1,2]. However, despite years of development and early-stage implementation, adoption in clinical practice remains inconsistent. Ethical, regulatory, and medicolegal uncertainties continue to limit the widespread use of AI in health care [3]. Physicians, as one of the primary users of these tools, face substantial uncertainty as they try to navigate the use of AI within systems where legal frameworks and professional guidelines have not kept pace with technological progress [4].

Previous studies have identified a wide range of barriers to AI adoption, including technical challenges, organizational obstacles, and cultural resistance within clinical environments [3]. Concerns about the accuracy, reliability, and robustness of AI-generated results remain major barriers to adoption in health care. While some models approach the diagnostic performance of physicians, their accuracy is often inconsistent [5]. Many AI tools lack validation in real-world settings, raising concerns about their generalizability and safety [6]. Physicians are particularly wary of the *black box* nature of machine learning [2,7], which obscures decision-making processes and hinders trust [8,9]. Concerns about algorithmic bias, data security, and the absence of transparent validation further contribute to clinicians’ hesitation to rely on these tools [7,10].

Legal and regulatory uncertainty continues to hinder AI adoption. In Canada, the lack of AI-specific legislation puts physicians in a situation where they must consider applying existing laws, including those related to civil liability, privacy, and human rights. Courts may continue to focus on traditional defendants (ie, clinicians and hospitals), potentially leaving health care providers responsible for AI-related failures [11]. Questions remain about patient consent, data sharing, and transparency obligations under current federal and provincial privacy laws, such as the federal Personal Information Protection and Electronic Documents Act and provincial and territorial privacy statutes. These laws apply broadly to the collection, primary use, secondary use, disclosure, and deidentification of personal health information, as well as to situations involving cross-border data transfers and third-party vendor contracts [1,4,12]. This fragmented framework increases compliance challenges, particularly for AI tools using cloud-based infrastructure located in other jurisdictions [3].

Although previous research has explored barriers to AI adoption primarily through general surveys and theoretical models [1,3], fewer studies have examined the practical, real-world challenges physicians face when AI is introduced into the day-to-day realities of medical practice. Little is known about the specific operational, legal, and professional dilemmas that arise at the point of care. This study addresses that gap by analyzing questions and concerns submitted by physicians to a medicolegal helpline, offering direct insight into the issues clinicians encounter in practice. Grounding our analysis in these real-world inquiries allows us to identify areas where policy, education, and AI development could better align with physicians’ legal obligations, professional standards, and patient safety.

## Methods

### Study Design and Setting

We conducted a thematic analysis of physician cases related to the use of AI in clinical practice, submitted to the Canadian Medical Protective Association’s (CMPA) medicolegal helpline between March 1, 2023, and February 28, 2025. The CMPA, a national medicolegal defense organization serving about 95% of practicing physicians in Canada, provides members with access to medicolegal advice and support through a dedicated helpline. During this period, CMPA physician advisors received 46 cases from members seeking guidance or raising concerns about the use of AI in their practice. These cases offered insights into physicians’ medicolegal and practice-related concerns and considerations regarding AI.

### Study Population

Practicing physicians who contacted the CMPA for advice on the use of AI in their practice during the study period were included in the study.

### Data Source

Data collection from CMPA cases has been described previously [2,13]. When physicians contact the CMPA, a physician advisor documents the purpose of the call and relevant information in a written memo. These memos are structured narrative summaries that capture the reason for the call, relevant clinical or practice context, and the physician’s questions or concerns, but they are not verbatim transcripts and contain no direct quotations. For this study, we analyzed memos from advice cases concerning the use of AI in clinical practice during the study period.

The unit of analysis was the coded medicolegal advice case, defined as 1 or more calls documented within a single advice file; therefore, the same physician could contribute more than 1 advice case over the study period. CMPA recorded 12,135 coded medicolegal advice cases during this period, of which 275 were coded as technology issues related to practice. Within this group, 46 cases met our definition of AI-related advice cases and were included in the analysis. AI-related cases were identified by reviewing physician advisor notes and selecting those in which the physician’s primary questions concerned AI tools used in clinical practice. For this study, we included only tools explicitly described as “artificial intelligence” (eg, AI scribes with speech recognition and generative summarization, large language model–based applications, or AI-supported decision tools) and excluded templating or basic speech-to-text systems without machine learning or generative components.

### Data Analysis

We conducted an inductive thematic analysis of advice case memos, following the approach described by Braun and Clarke [14]. Our goal was to identify themes related to physicians’ questions and concerns about the use of AI in clinical practice, as documented by physician advisors at the CMPA.

We attended to how physicians’ questions and perceived risks were described in the cases. For example, an excerpt in which a physician was “unsure whether they needed explicit patient permission before using the AI scribe” was coded as “uncertainty/question about consent process,” grouped under the subtheme “scope of consent for AI scribes,” within the broader theme “patient consent and communication.”

The data consisted of physician advisor summaries rather than verbatim transcripts and contained no direct quotations. Our analysis was based on the physicians’ questions and perceived risks, as documented in these second-order summaries.

One researcher (MM) conducted the initial coding of the entire dataset (46 cases) to develop a preliminary set of codes and build familiarity with the data. To support analytic calibration and clarify code definitions, a second researcher independently coded a subset (n=23, 50%) of cases. Following line-by-line comparison, percent agreement [14,15] was 97%, indicating a high level of concordance at this calibration stage. Coding differences were resolved through discussion and refinement of code definitions, resulting in a shared coding framework. The primary coder then recoded the full dataset using this refined framework to ensure consistent application across all cases. Throughout the process, codes were iteratively reviewed and grouped into subthemes and overarching themes through ongoing discussion between the researchers.

Theme labels were primarily data-led and then refined using terminology consistent with medicolegal and AI ethics literature. The primary coder had a background in engineering and public health, and the secondary coder had a background in science and biology; both had more than 3 years of work experience in a medicolegal organization. After the coding framework and themes were developed, we sought feedback from CMPA’s legal advisory board to ensure that the themes and subthemes were consistent with current medicolegal principles. This process led to minor wording adjustments but no substantive changes to the analysis.

### Ethical Considerations

The ethics review panel of the Advarra Institutional Review Board provided ethics approval for the conduct of this study (protocol number for secondary use of data 00020829). Before analysis, the memos were deidentified in accordance with organizational guidelines by removing or generalizing names, cities, and specific organizations (eg, referring to provincial regulatory authorities as “the College”). The medicolegal memos did not contain patient-identifiable information, and no patient-identifiable data were included in the research dataset. The deidentified data were then analyzed and reported only in aggregated form to minimize any residual risk of reidentification. Deidentified data were stored in CMPA’s secure data hub for a minimum of 10 years in accordance with CMPA’s retention and disposition policy. The compensation to participants was not applicable to this study, as it was a retrospective study. Members of the CMPA are informed at enrollment that their deidentified information may be used for internal research, with an option to opt out, and the Advarra Institutional Review Board approved this minimal-risk secondary use without requiring additional individual consent.

## Results

### Overview

A total of 46 AI-related cases were received by CMPA during the study period, with 22 (47.8%) cases in 2023 and 24 (52.2%) cases in 2024 (no AI-related cases were recorded in January 2025 or February 2025); in total, 40 (87%) cases were from family medicine specialists. Physicians contacted the service from multiple provinces, with most cases (n=18, 39.1%) originating from Ontario, Canada’s most populous province. This was followed by Quebec at 10 (21.7%), and British Columbia at 8 (17.4%). Alberta contributed 6 (13%) cases. Cases from Manitoba, Saskatchewan, New Brunswick, Nova Scotia, Newfoundland and Labrador, and the Northwest Territories of Canada together accounted for 4 (8.7%) AI-related advice cases.

### Thematic Analysis of the Advice Cases

**Figure 1.**
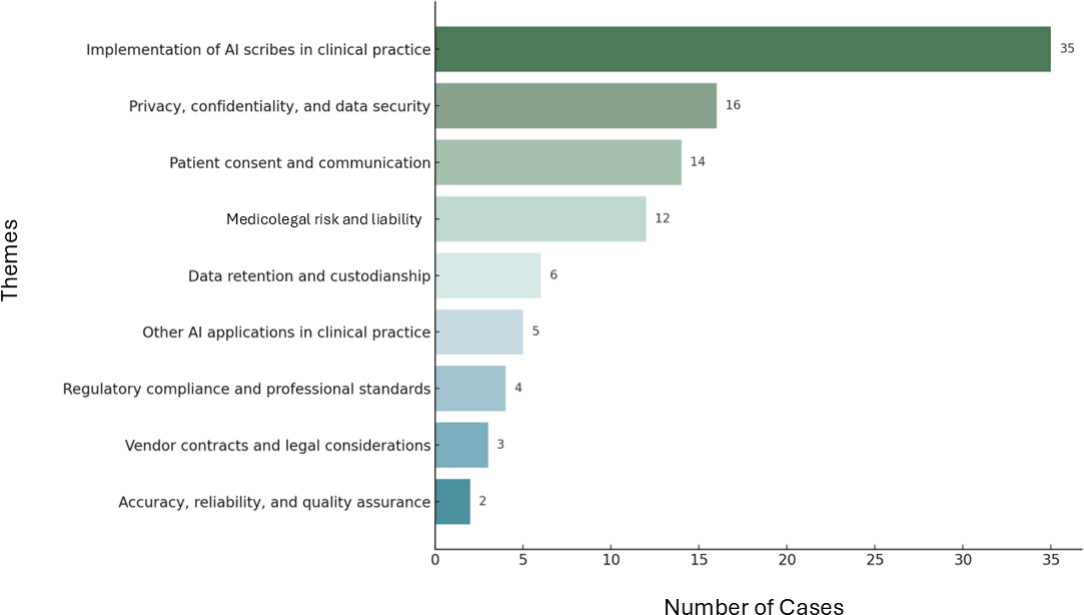
Themes and their frequency of occurrence in the dataset (N=46). AI: artificial intelligence.

The thematic analysis of the advice cases identified 8 distinct themes, reflecting physicians’ questions and concerns regarding the use of AI in clinical practice. These themes ranged from technical and regulatory considerations to ethical and medicolegal concerns. Figure 1 outlines each theme alongside its frequency of occurrence within the dataset. Several cases encompassed multiple themes, highlighting physicians’ concerns and uncertainty about navigating the medicolegal and practical challenges of using AI in clinical care.

### Implementation of AI Scribes in Clinical Practice

Physicians frequently sought guidance on integrating AI tools, particularly for scribing support, into their clinical workflows. Some inquired about using AI scribes to streamline note-taking and reduce the time spent on documentation. Others focused on clarifying the legal implications and professional accountability associated with AI-generated notes. There were also questions about potential risks, including inaccuracies in AI-transcribed records and the possibility of overreliance on automated documentation.

### Privacy, Confidentiality, and Data Security

Concerns about patient data protection and privacy requirements were a common theme. Physicians inquired about safeguarding patient information when using AI tools, especially when data handling involved servers or data processing activities located outside Canada. For example, some cases involved physicians developing AI tools who had questions about confidentiality and compliance with Canadian privacy laws. Other cases focused on privacy concerns related to the use of existing AI tools to manage electronic medical records (EMRs).

### Patient Consent and Communication

Physicians expressed uncertainty about the requirements for obtaining patient consent when using AI in clinical care. Questions addressed whether consent was necessary, whether it should be verbal or written, and how to proceed if consent had not been obtained. Some cases focused on the potential medicolegal risks of using AI tools without explicit consent, while others raised concerns about specific applications, such as AI-based transcription programs, and how these might affect the accuracy of medical records and related legal implications.

### Data Retention and Custodianship

Physicians raised questions about how AI-generated data should be stored and the appropriate length of retention. Inquiries sought clarification on data retention obligations and the risks of storing information on servers located outside Canada. Some cases questioned whether draft recordings or documents created outside the EMR were considered part of the medical record and whether physicians were required to provide them upon request. Others expressed concern about the risks of collecting and processing patient data on AI servers based in other countries.

### Regulatory Compliance and Professional Standards

Physicians sought clarification on how AI use aligned with professional standards and legal requirements. Questions included whether adherence to foreign laws or regulations, such as HIPAA (Health Insurance Portability and Accountability Act), met their obligations under Canadian law and whether internal processes and contracts met the expectations of regulatory bodies such as provincial and territorial medical regulatory authorities (Colleges). For example, physicians questioned whether a HIPAA-compliant, AI-supported Subjective, Objective, Assessment, and Plan note system would also meet Canadian privacy legislation requirements. Others inquired whether their user agreements for developing AI tools for clinical use were sufficient to meet applicable regulatory requirements. Physicians raised concerns about both electronic health record–embedded AI tools and stand-alone software as a service products used alongside the EMR; however, the memos did not provide enough detail to systematically distinguish concerns by vendor type, and callers did not reference specific provincial or College guidance by name.

### Medicolegal Risks and Liability

Physicians expressed concerns about potential legal risks associated with AI tools, including the possibility of negligence claims, College complaints, and broader medicolegal exposure. Some sought advice after developing AI-like tools, asking whether their use could create legal liability. Others raised concerns about the use of AI software in walk-in clinics to gather patient information and the associated medicolegal implications.

### Vendor Contracts and Legal Considerations

Physicians questioned whether they should engage legal authorities to review contractual terms and ensure compliance with privacy and professional standards. Physicians wanted to ensure that their template legal agreement adequately addressed risks associated with processing patient data. Some, eager to implement an AI service, sought guidance on resources for evaluating a vendor’s contract and ensuring its legal soundness.

Within this theme, physicians most often sought advice on whether their contracts needed to address the following: who would own and be custodian of both source and AI-generated data; where data would be stored or processed, including whether cross-border transfers created additional medicolegal risk; how long data would be retained and under what conditions it would be deleted or returned; whether, and to what extent, they should be concerned about third-party access to the data and the safeguards governing such access; and whether provisions allocating responsibility and indemnity would adequately protect them in the event of a privacy breach or AI-related error. These questions were framed in terms of how such clauses might affect the physician’s medicolegal risk.

### Accuracy, Reliability, and Quality Assurance

Some physicians expressed concerns about the accuracy and reliability of AI-generated documentation, including the potential for misinterpretation or bias in clinical notes. Physicians sought guidance on using an AI-assisted application for clinical notes and raised specific concerns about the risks of misinterpretation or bias in AI-generated transcriptions. Although this concern appeared less frequently, it highlights physicians’ awareness of the clinical risks associated with overreliance on AI tools.

## Discussion

### Principal Findings

This qualitative analysis of physicians’ inquiries to the CMPA provides valuable insight into the uncertainties and perceived risks clinicians face when considering the adoption of AI tools in clinical practice. While physicians recognized the potential of AI to streamline documentation, improve workflow efficiency, and support various aspects of patient care, they also voiced significant concerns about privacy, patient consent, data governance, and medicolegal liability. Compared with existing medicolegal literature, our findings both confirm and extend previous work. Survey and interview studies show that clinicians are broadly concerned about medicolegal liability, privacy risks, and the possibility of becoming “liability sinks” when using AI tools [11,16]. Our real-world data support these concerns but also provide more operational detail than previous studies. Rather than expressing only general worries, physicians asked concrete questions about contractual vulnerabilities, such as whether vendor agreements adequately addressed cross-border data processing, indemnity, and compliance with Canadian privacy law. This level of detail has not been reported in previous clinician surveys or qualitative interviews and represents a key contribution of this practice-based dataset [11,16].

Our analysis found that implementation-related inquiries dominated the cases, particularly those focusing on AI scribes. This suggests a growing interest in solutions to mitigate administrative burden, aligning with recent evidence indicating that AI scribes can alleviate documentation workload and reduce physician burnout. A 2024 rapid review reported reductions in both mental demand and frustration when AI scribes were used, suggesting tangible benefits for clinical efficiency [17]. Despite this promise, the cases in our study reflected physicians’ persistent uncertainty about the practical steps of implementation, including how to integrate AI into existing workflows and how to manage potential medicolegal implications. These concerns were also acknowledged in the same review, which emphasized the need for thoughtful planning, clinician training, and technical support to ensure safe and effective AI integration [17]. Our findings also diverge from the literature regarding consent for AI scribes. Although several Colleges now mandate informed consent for AI-supported documentation, previous studies have not explored how clinicians implement these requirements in practice.

Patient-centered research shows that patients expect to be informed about AI use and may view such disclosures as equally important as traditional risk discussions [18,19]. However, patient perspectives are shaped by factors such as gender, age, income, and health status [18,20], complicating efforts to establish standard consent processes and highlighting the need for tailored communication. Many medical regulatory bodies, including the College of Physicians and Surgeons of Nova Scotia, mandate consent for recordings involving AI scribes [21]. However, despite emerging guidance, gaps remain in defining comprehensive consent practices for AI, particularly as patient apprehensions about safety, choice, and data security persist [19]. Our findings highlight ongoing uncertainty among physicians and the critical need for clearer, practice-oriented frameworks to support consent in AI integration, suggesting a gap between regulatory expectations and frontline understanding that has not been described in previous medicolegal or implementation studies [18,22].

Physicians’ cases also demonstrated concern regarding privacy and data security, particularly about the handling of patient information by AI systems, cross-border data transfers, and reliance on servers outside Canada. Comparative analyses of privacy frameworks in Canada, the United States, and other jurisdictions show cross-border data flow as a critical regulatory issue [18]. Furthermore, physicians’ distinction between the US HIPAA compliance and Canadian privacy law highlights their nuanced understanding of professional and legal obligations and the limitations of relying solely on international frameworks [23]. However, there remains a need for clear guidelines and policies to address similar problems.

Questions about data governance and custodianship revealed areas of uncertainty, particularly concerning the legal status of draft notes and recordings generated by AI systems. While existing literature discusses data governance at a high level, it does not address whether transient AI-generated artifacts constitute part of the medical record or trigger retention obligations.

Physicians were unclear about whether such records fall under their record-keeping responsibilities, an ambiguity that reflects wider regulatory gaps in Canadian health care. Existing analyses of AI regulation in Canada have highlighted that, alongside safety considerations, AI technologies raise unresolved questions about data ownership and retention [23,24]. Our analysis surfaces these previously underreported uncertainties, highlighting the added value of real-time physician inquiries in identifying emergent medicolegal challenges not visible in survey-based or interview-based studies.

Medicolegal risk and liability associated with AI systems emerged as another prominent theme, with physicians expressing concern about responsibility for AI-related errors and regulatory breaches. Although current case law provides limited guidance, the potential for legal exposure remains a significant deterrent to adoption. Our cases reflected a focus on medicolegal risks rather than technical issues (eg, reliability or accuracy), likely because the CMPA is a medicolegal organization. Our results suggest that legal and operational uncertainties are a barrier to AI integration in physicians’ clinical practice. Most of the cases in our study came from family medicine specialists, and most of the AI tools they sought advice about were administrative rather than clinical, such as AI scribes. For this reason, themes centered more on issues related to patient privacy and consent for the use of AI tools than on issues related to diagnostic performance. We observed only a few cases about diagnostic AI technologies and issues related to diagnostic accuracy, reliability, and bias, which may be due to fewer cases from specialties such as radiology in our dataset. We believe this focus is reflective of the types of cases we received and may not be generalizable. Nevertheless, issues related to diagnostic accuracy, reliability, and bias remain critical and should be addressed through regulation and standardization as AI becomes more integrated into health care.

Addressing these concerns requires coordinated action. Physicians and health care institutions should establish formal evaluation processes for AI tools, ensuring that privacy protections, consent mechanisms, and data governance policies are firmly in place before adoption [25]. Communication strategies should be developed to help physicians explain AI use clearly to patients, with sensitivity to demographic variations in preferences [18].

Regulatory bodies have a pivotal role in supporting safe AI adoption. Clear, AI-specific privacy guidelines should address the complexities of cross-border data transfers and third-party data access. Standardized consent frameworks are also essential, balancing patient autonomy with practical clinical workflows. Moreover, regulatory interpretations of safety must expand to encompass AI-specific risks, including algorithmic bias and opaque decision-making processes. Educational resources should be developed to guide physicians in understanding how AI use intersects with existing professional obligations [26].

For technology developers, there should be a clear mandate to design AI tools that align with Canadian privacy law and support physicians’ ethical and professional responsibilities. Features that document consent, ensure transparent data handling, and provide robust error detection will be critical to facilitating safe integration into clinical practice.

In summary, our study is among the first exploratory efforts to highlight the multifaceted concerns of Canadian physicians as they navigate the complexities of AI adoption in health care, grounded in their real, day-to-day experiences. It identifies gaps in guidance and technology design essential to support the responsible integration of AI into clinical practice. Our findings suggest that, before adopting AI scribes or similar tools, physicians and organizations should consider the following governance questions: who is the data controller or custodian for both source and AI-generated data; what types of data are retained (eg, audio files, draft transcripts, and final notes) and for how long; where data are stored and processed, including any cross-border transfers; how patient consent is obtained and evidenced in the record (verbal vs written); how errors, bias, reliability, and accuracy in AI outputs are monitored and addressed; and which contractual clauses (eg, privacy safeguards, breach notification, data return or destruction, and indemnity) are in place to mitigate medicolegal risk.

### Limitations and Future Directions

While this study provides timely insights into physicians’ concerns about AI adoption, it is limited to inquiries submitted to a single national medicolegal advisory service and predominantly from family medicine specialists. As such, it may not fully capture the dynamics in larger institutions, such as hospitals or health systems, where technology adoption decisions are often made at an organizational level rather than by individual practitioners. Most advice cases concerned documentation-focused AI scribes and related administrative tools, and advisor memos did not consistently provide sufficient technical detail to distinguish transcription-only systems from AI scribes with generative functions. Because the data consist of advisor-written summaries rather than verbatim transcripts, medicolegal documentation conventions may foreground risk and liability and underrepresent clinical nuance or emotional tone, which may have influenced the themes identified. Given that our dataset included only AI-related concerns, future research could compare AI-related inquiries with non-AI software inquiries (eg, image-recording or EMR systems) to assess whether consent and privacy issues differ across technologies. Future work could also examine a broader range of physicians’ real-world experiences with AI implementation, as well as patient perspectives on AI use in health care settings. Longitudinal research would be valuable in tracking how physicians’ concerns evolve with increasing familiarity and use of AI tools. Additionally, further exploration of how regulatory frameworks adapt to the unique challenges of AI in health care will be essential for informing safe, ethical, and effective technology integration.

## Abbreviations

AI: artificial intelligence
CMPA: Canadian Medical Protective Association
EMR: electronic medical record
HIPAA: Health Insurance Portability and Accountability Act
